# Noncovalent Grafting of Molecular Complexes to Solid Supports by Counterion Confinement

**DOI:** 10.1021/acs.jpcc.3c05691

**Published:** 2023-12-12

**Authors:** Petrus C. M. Laan, Eduard O. Bobylev, Norbert J. Geels, Gadi Rothenberg, Joost N. H. Reek, Ning Yan

**Affiliations:** † Van’t Hoff Institute for Molecular Sciences, University of Amsterdam, Science Park 904, Amsterdam 1098 XH, The Netherlands; ‡ Key Laboratory of Artificial Micro- and Nano-Structures of Ministry of Education, School of Physics and Technology, Wuhan University, Wuhan 430072, China

## Abstract

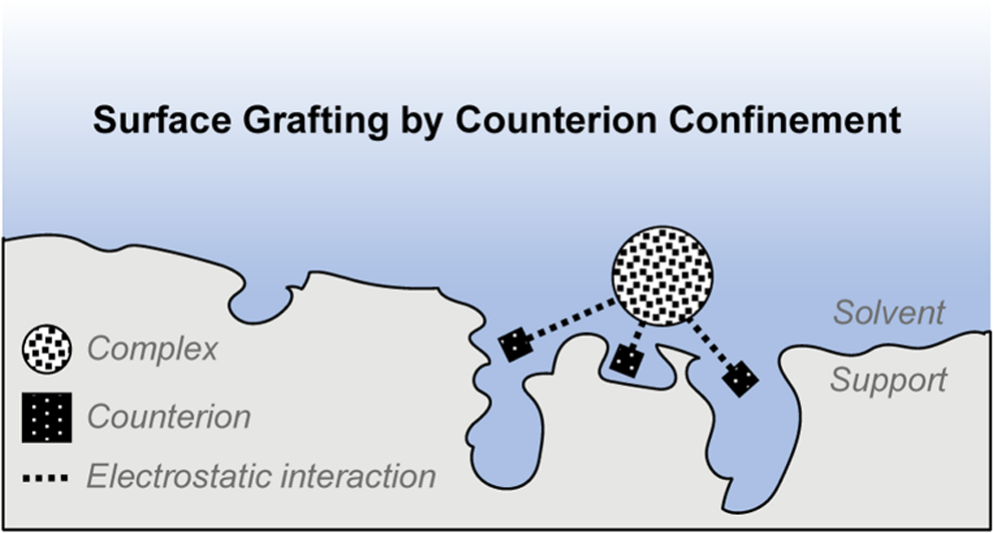

Grafting molecular complexes on solid supports is a facile strategy to synthesize advanced materials. Here, we present a general and simple method for noncovalent grafting on charge-neutral surfaces. Our method is based on the generic principle of counterion confinement in surface micropores. We demonstrate the power of this approach using a set of three platinum complexes: **Pt_1_** (Pt_1_L_4_(BF_4_)_2_, L = *p*-picoline), **Pt**_2_ (Pt_2_L_4_(BF_4_)_4_, L = 2,6-bis(pyridine-3-ylethynyl)pyridine), and **Pt_12_** (Pt_12_L_24_(BF_4_)_24_, L = 4,4′-(5-methoxy-1,3-phenylene)dipyridine). These complexes share the same counterion (BF_4_^–^) but differ vastly in their size, charge, and structure. Imaging of the grafted materials by aberration-corrected high-angle annular dark-field scanning transmission electron microscopy (AC-HAADF-STEM) and energy-dispersive X-ray (EDX) showed that our method results in a homogeneous distribution of both complexes and counterions. Nitrogen sorption studies indicated a decrease in the available surface area and micropore volume, providing evidence for counterion confinement in the surface micropores. Following the adsorption of the complexes over time showed that this is a two-step process: fast surface adsorption by van der Waals forces was followed by migration over the surface and surface binding by counterion confinement. Regarding the binding of the complexes to the support, we found that the surface–adsorbate binding constant (*K*_S_) increases quadratically with the number of anions per complex up to *K*_S_ = 1.6 × 10^6^ M^–1^ equaling Δ*G*°_ads_ = −35 kJ mol^–1^ for the surface binding of **Pt_12_**. Overall, our method has two important advantages: first, it is general, as you can anchor different complexes (with different charges, counterions, and/or sizes); second, it promotes the distribution of the complexes on the support surface, creating well-distributed sites that can be used in various applications across several areas of chemistry.

## Introduction

Addressing today’s environmental challenges requires a redesign of the energy and chemical sectors.^1^ The rate of this change depends on the development of new functional materials for electrolyzers,^2^ solar-to-fuel processes,^3^ and metal-air batteries.^4^ The energy transition will only take place if we can provide economically viable alternatives.^5^ Carbon-based catalytic materials are especially important here, owing to their low cost, high thermal and electrical conductivity, and tunable surface properties.^6−8^

Advancing these materials further often requires a combination of functionalities such as catalytic activity and electrical conductivity. This can be done by anchoring molecular complexes on carbon-based supports, most commonly by covalent binding.^9^ Yet this method incurs additional steps, increasing the cost and hampering large-scale application. Moreover, the covalent bonds can change the function of the molecular complex and typically require custom-made solutions for each case.

A much simpler approach is grafting the complexes on surfaces using sorption methods, but these bindings are often too weak.^10^ One way to solve this is by immobilization based on electrostatic interactions, where weakly coordinating counterions are exchanged by a charged solid that acts as a “counterion” instead. This method, however, works only for charged supports.^11^

Here, we describe a new and general strategy for immobilizing molecular species on charge-neutral carbon-based materials. Our concept is based on noncovalent anchoring, by confining the counterions of the complexes in the support pores. We test this concept by immobilizing a series of platinum complexes with varying charges on porous carbon. The anchored complexes were characterized by aberration-corrected high-angle annular dark-field scanning transmission electron microscopy (AC-HAADF-STEM), energy-dispersive X-ray (EDX) imaging, and detailed nitrogen sorption studies. Our results show that the counterions are indeed confined in the micropores and that the complexes are evenly distributed over the support. Adsorption kinetic studies showed that this immobilization is a two-step process. Moreover, the adsorption isotherms showed that the binding is stronger as the number of counterions per complex increases. This general yet simple method opens opportunities for making new functional materials for the Energy Transition.

## Experimental Section

### General Considerations

All reactions were carried out in air at room temperature, unless noted otherwise. To prevent cross-contamination of trace metals, all glassware used were single-use scintillation vials or glassware which were cleaned with aqua regia (HNO_3_/HCl 1:3 molar ratio) before use. All water used was demineralized water, which was deionized by the Milli-Q technique and has a resistance greater than 18.2 MΩ cm at room temperature. All reagents were purchased from commercial suppliers and used as received, unless mentioned otherwise. Specifically, Vulcan XC72 was obtained from Cabot and acetonitrile (99.9%, Extra Dry over Molecular Sieve, AcroSeal) was obtained from Fisher Scientific B.V. The self-assembled supramolecular structures Pt_1_L_4_(BF_4_)_2_ (**Pt**_**1**_, L = *p*-picoline),^12^ Pt_2_L_4_(BF_4_)_4_ (**Pt**_**2**_, L = 2,6-bis(pyridine-3-ylethynyl)pyridine),^13^ Pt_2_L_4_(BArF)_4_ (**Pt**_**2**_(BArF), L = 3,3′-((5-methoxy-1,3-phenylene)bis(ethyne-2,1-diyl))dipyridine, BArF = [{3,5-(CF_3_)_2_C_6_H_3_}_4_B]),^12^ and Pt_12_L_24_(BF_4_)_24_ (**Pt**_**12**_, L = 4,4′-(5-methoxy-1,3-phenylene)dipyridine)^14^ were synthesized according to literature procedures. **Pt**_**1**_ was stored as a solid, and **Pt**_**2**_, **Pt**_**2**_(BArF), and **Pt**_**12**_ were stored as solutions (5.0 mM Pt) in J Young Schlenk flasks at 5 °C.

### Instrumentation and Characterization Methods

N_2_ adsorption–desorption isotherms were measured on a Thermo Scientific Surfer instrument at 77 K, using vacuum-dried samples. More specifically, around 100 mg of each sample was dried at 100 °C for 16 h on a Belprep-vacIII prior to analysis. The specific surface area (SSA) was determined based on the adsorption branch, and the Brunauer–Emmett–Teller (BET) analysis was performed according to the Rouquerol consistency criteria.^15−17^ All samples satisfied the four Rouquerol criteria (Figures S1–S7). The pore size distributions (PSDs) were determined based on the adsorption branch of the isotherm using the Horvath–Kawazoe (HK) technique^18^ including the additions of Saito and Foley.^19^ We refrained from using (N)L-DFT type of models to avoid model-induced artifacts and user-input dependent results.^20^ The H–K method is known to yield more reliable PSDs in the micropore range for carbon-based materials.^21^

Aberration-corrected high-angle annular dark-field scanning transmission electron microscopy (AC-HAADF-STEM) measurements, elemental mappings, and energy-dispersive X-ray spectroscopy (EDS) measurements were taken on a Thermo Fisher Scientific FEI Themis Z instrument coupled with a high-angle annular dark-field (HAADF) detector and an energy-dispersive X-ray spectroscopy detector. The samples were deposited on a Holey carbon-coated TEM-grid (300 mesh Cu grid, Pacific Grid Tech). The setup was operated at 200 kV and delivered a spatial resolution of ≤70 pm in both TEM and STEM, resulting in atomic-resolution imaging of the samples.

UV–vis spectra were obtained on a double-beam Shimadzu UV-2600 spectrometer at room temperature by using quartz cuvettes with a path length of 2 or 10 mm and clean solvent as a background. Spectra were obtained between 225 and 500 nm by using a spectral bandwidth of 0.5 nm. After data acquisition, the spectra were zeroed at 500 nm.

Metal loadings were determined based on inductively coupled plasma optical emission spectrometry (ICP-OES) analysis (Mikroanalytisches Laboratorium Kolbe, Oberhausen, Germany). Samples were prepared using microwave digestion and then analyzed with a Spectro Arcos analyzer of Spectro.

### Surface Immobilization

The self-assembled supramolecular structures **Pt**_**1**_, **Pt**_**2**_, and **Pt**_**12**_ were immobilized following a published procedure.^22^ In short, Vulcan (150 mg) was finely dispersed as a black suspension in acetonitrile (11.25 mL (for 5.0 μmol of Pt g_vulcan_^–1^) or 9.00 (for 12.5 μmol of Pt g_vulcan_^–1^)) by ultrasonication in a 100 mL round-bottom flask for 1 h. Then, an acetonitrile solution of the respective organometallic complex (1.5 mL, 0.5 mM Pt, 0.75 μmol of Pt (for 5.0 μmol of Pt g_vulcan_^–1^)) or 3.75 mL, 0.5 mM Pt, 1.875 μmol of Pt (for 12.5 μmol of Pt g_vulcan_^–1^) was added dropwise to the Vulcan suspension over the course of 3 h under vigorous stirring. After complete addition, the reaction mixture was left to stir at room temperature for an additional 16 h. Thereafter, acetonitrile was removed *in vacuo* over the course of 1 h. The remaining black powder was further dried under vacuum (5 mbar at 50 °C) for 16 h and named **Pt**_**1**_/Vulcan, **Pt**_**2**_/Vulcan, and **Pt**_**12**_/Vulcan, respectively.

### Surface Desorption

The stability of the hybrid materials was tested by desorption experiments. **Pt**_**2**_/Vulcan was finely dispersed as a black suspension in MeCN or DMSO by ultrasonication in a screw-cap vial and stirred for 16 h at room temperature. Another sample of **Pt**_**2**_/Vulcan was subjected to Soxhlet extraction with MeCN for 16 h. Hereafter, the reaction mixtures were centrifuged (4000 rpm for 5 min) giving a black residue and a colorless to slightly yellow supernatant. The supernatant was subsequently analyzed by UV–vis spectroscopy to quantify the amount of desorbed material (Table S2).

### Adsorption Kinetics

The adsorption kinetics of **Pt**_**1**_, **Pt**_**2**_, **Pt**_**2**_(BArF), and **Pt**_**12**_ on the heterogeneous support Vulcan were followed over time by UV–vis spectroscopy via the solution depletion method. The ratios between support, solvent, and the adsorbate were kept identical to these as used during complex immobilization (5.0 μmol Pt g_vulcan_^–1^). Thus, Vulcan (286 mg) was finely dispersed as a black suspension in acetonitrile (19.71 mL) by ultrasonication in a 100 mL round-bottom flask for 1 h. Then, an acetonitrile solution of **Pt**_**1**_, **Pt**_**2**_, **Pt**_**2**_(BArF),or **Pt**_**12**_ (286 μL, 5.0 mM Pt, 1.43 μmol of Pt) was added instantaneously to the Vulcan suspension under vigorous stirring. While the reaction mixture was left to stir at room temperature for 16 h, seven samples (1.5 mL of the suspension) were taken at 15 s, 1 min, 4 min, 16 min, 64 min, 2 h, and 16 h. After filtration of the suspension over a 0.45 μm filter, the filtrates were analyzed by UV–vis spectroscopy. The decrease in adsorption was attributed to immobilization of the molecular species on the heterogeneous support Vulcan. The remaining concentrations of the molecular species samples in the filtrates were determined using their respective UV–vis calibration curves (Figures S9–S14).

### Adsorption Isotherms

The adsorption isotherms of **Pt**_**1**_, **Pt**_**2**_, and **Pt**_**12**_ on the heterogeneous support Vulcan were determined by UV–vis spectroscopy via the solution depletion method using eight different concentrations. Vulcan (57.2 mg) was finely dispersed as a black suspension in acetonitrile (3.440–3.942 mL) by ultrasonication in a 15 mL centrifuge tube for 1 h. Then, acetonitrile solutions (0.5 mM Pt) of **Pt**_**1**_, **Pt**_**2**_, or **Pt**_**12**_ (0.560, 0.280, 0.215, 0.166, 0.127, 0.098, 0.075, and 0.058 mL) were added instantaneously to the Vulcan suspensions so that the total volume in all samples was equal (4.00 mL). The samples were equilibrated using a temperature-controlled shaking water bath (Julabo SW23) at 200 rpm at 22 °C for 10 min, 2 h or 16 h. Afterward, the samples were centrifuged (5000 rpm for 10 min) resulting in a black residue and a yellow to colorless supernatant, which was analyzed by UV–vis spectroscopy. The decrease in adsorption was attributed to immobilization of the molecular species on the heterogeneous support Vulcan. The remaining concentration of the molecular species samples in the filtrates was determined using their respective UV–vis calibration curves (Figures S9–S14). The adsorption data was fitted to the solution analogue of the Brunauer–Emmett–Teller model using Origin 2018 (eq 1).^23^
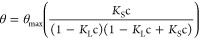
1where θ is the total surface coverage in μmol_adsorbate_ g_Vulcan_^–1^, θ_max_ is the monolayer surface coverage in μmol_adsorbate_ g_Vulcan_^–1^, *K*_S_ is the surface–adsorbate binding constant in M^–1^, *K*_L_ is the adsorbate–adsorbate binding constant in M^–1^, and *c* is the equilibrium adsorbate concentration in M.

## Results and Discussion

### Design and Synthesis of the Model System

**Figure 1:**
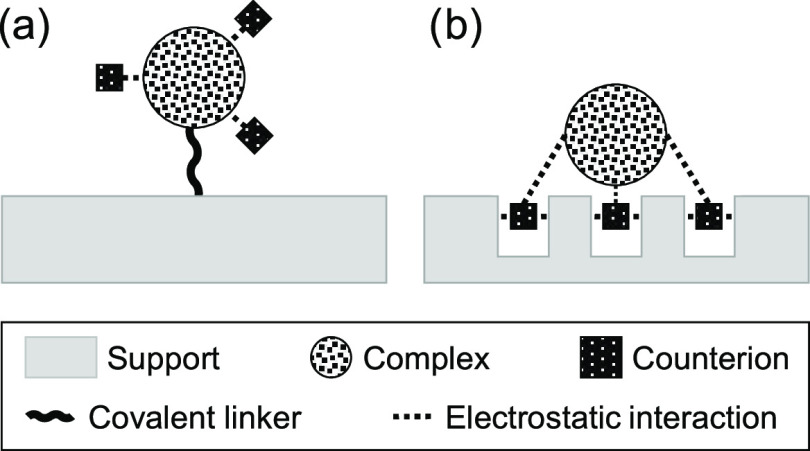
Methodologies to perform immobilization by (a) covalent tethering and (b) noncovalent grafting by counterion confinement.

Our hypothesis was that confining the counterions in the surface pores would result in strong binding of the complex to the support surface, especially if three criteria are met: (i) the complex has multiple counterions, (ii) the counterions fit well in the pores, and (iii) additional stabilizing interactions between the counterion and pore are possible (Figure 1).

**Figure 2:**
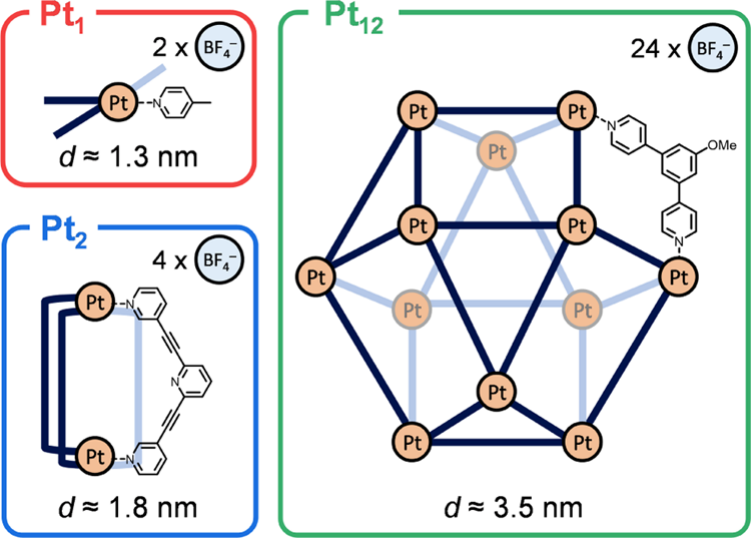
Ball-and-stick model structures of the three complexes **Pt_1_**, **Pt**_**2**_, and **Pt_12_**. The balls represent Pt atoms, and the sticks represent the connecting organic moieties (one of which is shown for each structure).

To test this hypothesis, we prepared a set of three platinum complexes {**Pt**_**1**_, **Pt**_**2**_, and **Pt**_**12**_} shown in Figure 2. These complexes, which were synthesized following published procedures,^12−14^ vary in their charge from [^2+^] to [^24+^], yet share the same anion [BF_4_^–^]. This set allows us to study the dependence of surface binding on the number of anions per complex (criterion 1) and the application to complexes of various sizes. As a support, we chose Vulcan XC72 (hereafter: Vulcan) owing to its high surface area (∼210 m^2^ g^–1^) and abundant micropores (∼15% of *V*_tot_) for confining the counterions (criterion 2).^24^ Besides these physical properties, Vulcan is an interesting substrate because of its widespread application in electrochemistry due to its high conductivity. Moreover, the BF_4_^–^ counterions could form stabilizing anion–π interactions with the graphitic π-systems of Vulcan (criterion 3).^25^ These graphitic π-systems are common in (activated) (nano) carbons and two-dimensional (2D) structures as graphene.^26,27^ The anion–π interaction can in principle also happen on the graphitic surface itself, yet it is more pronounced in surface pores as the interaction can be from multiple sites.

The surface immobilization of the complexes on Vulcan (denoted hereafter as **Pt**_**1**_/Vulcan, **Pt**_**2**_/Vulcan, and **Pt**_**12**_/Vulcan) was done using conventional wet impregnation from a MeCN solution (see further details in the Experimental Section). In each case, we prepared samples with two Pt loadings: 5.0 and 12.5 μmol of Pt g_vulcan_^–1^, respectively. Choosing for loading by weight rather than by molarity enables a comparison across the set, as otherwise, the total number of anions per sample would differ by an order of magnitude. This also ensures an identical number of counterions among samples of the same loading as the Pt/BF_4_ ratio of 1:2 for all compounds. Furthermore, by preparing different loadings of each sample, we could study trends correlated with platinum loading.

### Physical Characterization of the Immobilized Complexes

**Figure 3:**
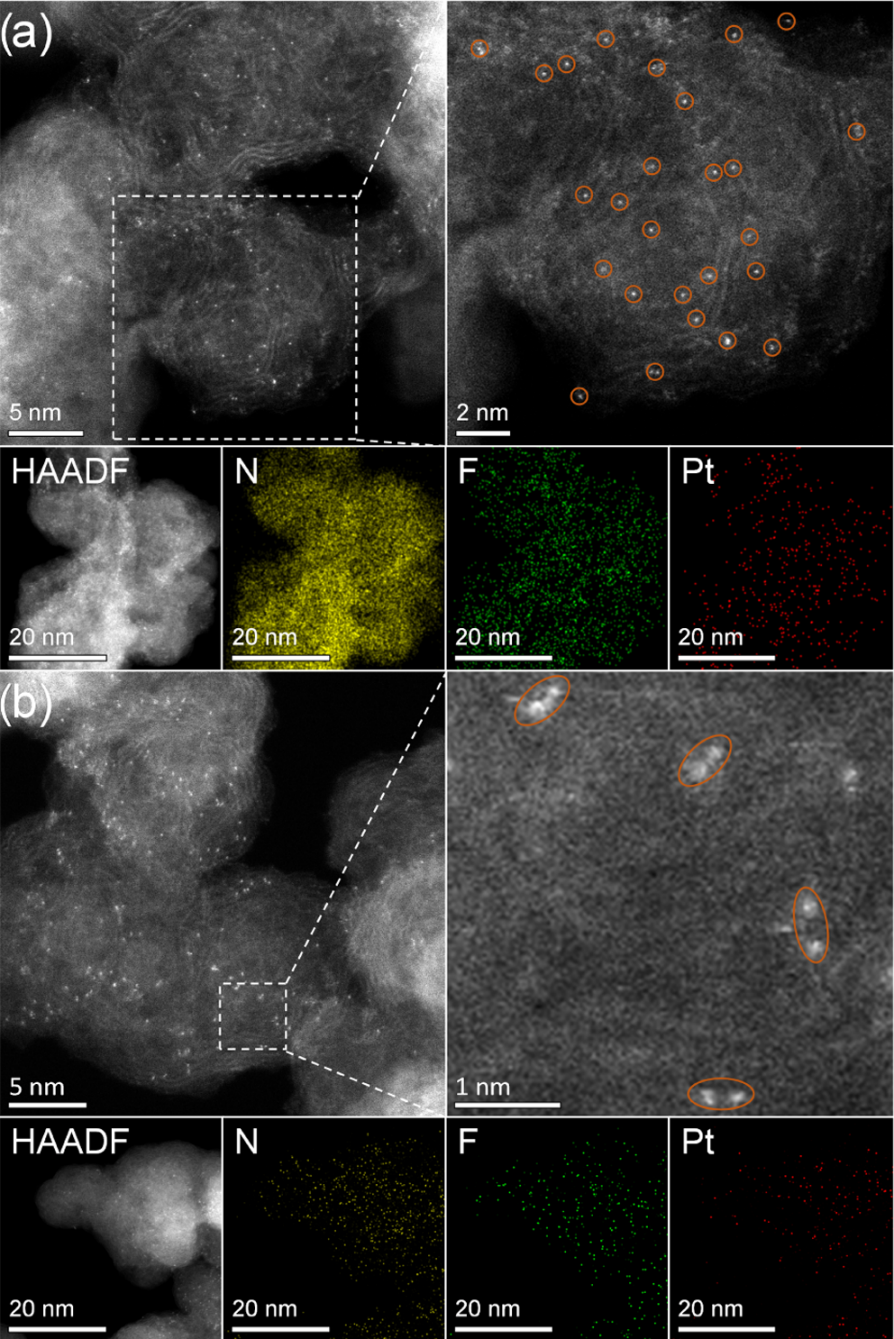
AC-HAADF-STEM-EDX imaging of (a) **Pt_1_**/Vulcan and (b) **Pt_2_**/Vulcan (5.0 μmol Pt g_Vulcan_^–1^). AC-HAADF-STEM imaging (left panels) and zoom-ins (right panels) showing single bright dots of Pt atoms as indicated by the orange circles and ovals. The EDX mapping shows the equal distribution of N, F, and Pt over the surface of the samples. The variation in the EDX intensity between samples is attributed to different acquisition parameters.

The impregnated complexes were characterized by by aberration-corrected transmission electron microscopy and energy-dispersive X-ray (EDX) imaging to study the distribution of the complexes and their counterions over the Vulcan surface (Figure 3). Both techniques showed that the complexes and counterions were homogeneously distributed over the surface without any clustering. This means that our impregnation method ensures a direct interaction between the support and all complexes, which is critical for combining the useful functionalities of the support and complex in designing advanced materials. The loading was determined by ICP-OES, which was in perfect agreement with the intended loading of 5.0 μmol of Pt g_Vulcan_^–1^ (Table S1).

The stability of the immobilized complexes was assessed by multiple desorption studies using **Pt**_**2**_/Vulcan at a 5.0 μmol Pt g_Vulcan_^–1^ loading (see further details in the Experimental Section). We first tried to desorb **Pt**_**2**_ using MeCN, the solvent used during impregnation. Neither a simple extraction at room temperature nor a Soxhlet extraction yielded any desorption of **Pt**_**2**_, indicating strong surface binding and thermal stability (Table S2). **Pt**_**2**_ could quantitively be desorbed by extraction with DMSO at room temperature.^22^

**Figure 4:**
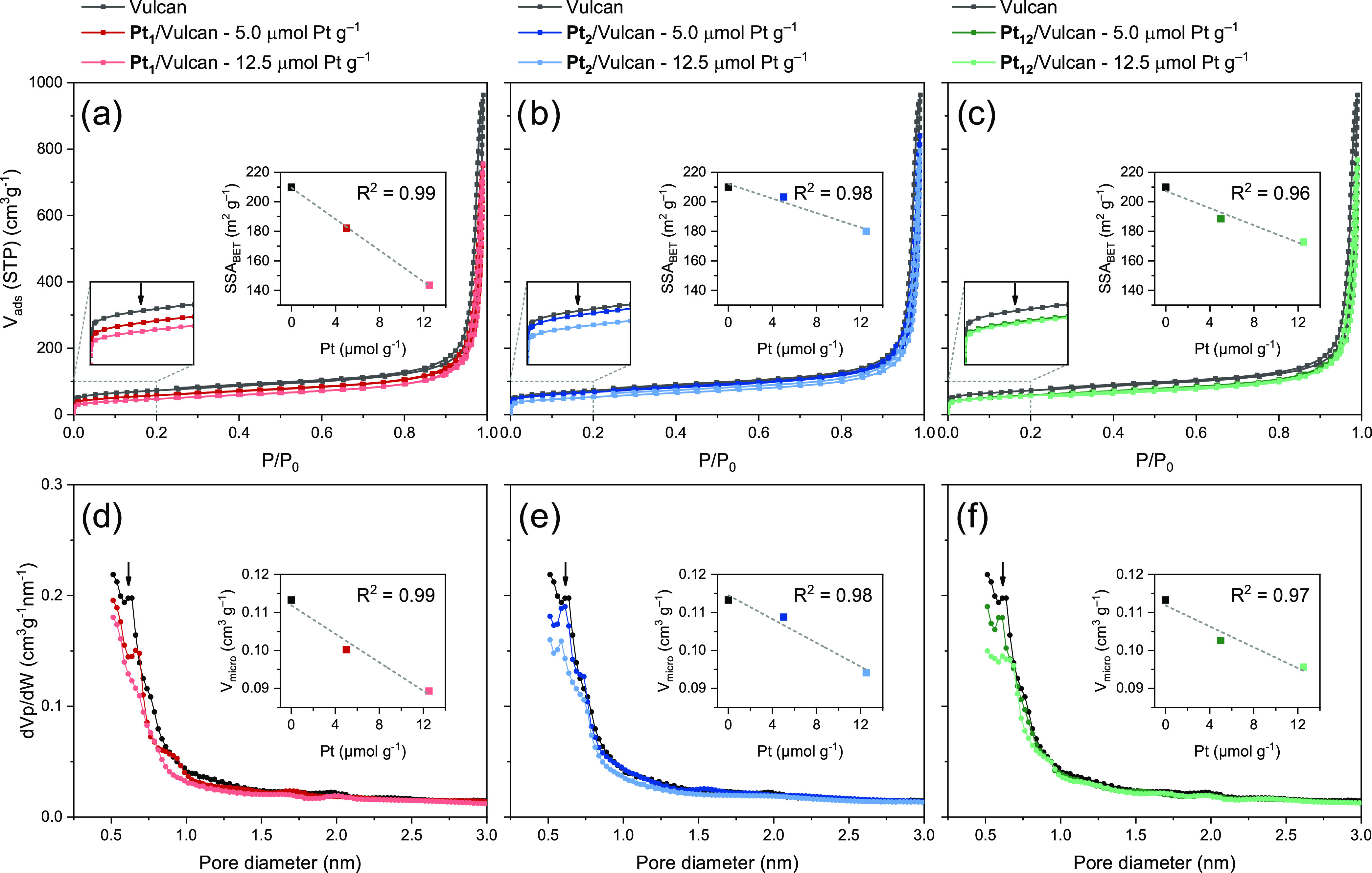
Physical characterization of **Pt_1_**/Vulcan, **Pt_2_**/Vulcan, and **Pt_12_**/Vulcan at different platinum loadings by N_2_ adsorption at 77 K. (a–c) N_2_ adsorption isotherms at 77 K. Insets show a linear fit between the specific surface area (SSA) obtained using the Brunauer–Emmett–Teller (BET) method and the platinum loading. (d–f) The corresponding micropore size distributions using the Horváth–Kawazoe method. Insets show a linear fit between the total micropore volume (*V*_micro_) and platinum loading.

The surface area and porosity of the grafted materials were studied using N_2_ adsorption at 77 K (Figures 4 and S1–S7, Table S3). The sorption isotherms are all highly similar and show classical type II curves indicating microporous materials with cylindrical pores.^15^ They do differ in two aspects: the total adsorbed volume and the adsorbed volume at low partial pressures (Figure 4a–c).

These results tell us two things: First, the decrease in total adsorbed volume is in line with the respective SSAs and comes with an increasing complex loading (see insets in Figure 4a–c). All of the samples show that there is less surface area accessible for N_2_ adsorption after immobilizing **Pt**_**1**_, **Pt**_**2**_, or **Pt**_**12**_ on Vulcan. This cannot be explained by simply covering the surface with complexes because the decrease in surface area is a factor of 5 higher than what would be expected based on the actual size of the complexes (Table S4 and further details in the Supporting Information).^28,29^ A more reasonable explanation is pore blocking by counterion confinement or adsorbed complexes covering pore entrances (*vide infra*). The fact that the decrease is linear (see insets in Figure 4a–c) also supports the submonolayer dispersed adsorption of **Pt**_**1**_, **Pt**_**2**_, and **Pt**_**12**_. Indeed, this is also what we see in the AC-HAADF-STEM images in Figure 3 for **Pt**_**1**_ and **Pt**_**2**_.

Second, the steep rise at low partial pressures is less pronounced with increasing complex loadings and is seen in all three cases (see the zoomed-in view in Figure 4a–c). This indicates that fewer micropores are available for nitrogen sorption after immobilization. We determined the micropore size distributions using the Horvath–Kawazoe method including the additions of Saito and Foley to describe the cylindrical pores properly too (see further details in the Experimental Section). We see a decrease in pores with a diameter of ∼0.7 nm, independent of which complex is immobilized (Figure 4d–f). As the complexes’ hydrodynamic radii are >0.7 nm, this decrease cannot be due to the confinement of the complexes in the surface micropores. The BF_4_^–^ counterions, however, are only ∼0.3 nm in diameter and fit well into these pores, thereby satisfying criterion 2. Moreover, the optimal distance for stabilizing anion–π interactions between BF_4_^–^ and graphitic π-systems is ∼0.35 nm (measured from the boron atom to the π-system).^25^ This is exactly half of the decreasing pore diameter, indicating a perfect fit of BF_4_^–^ anions with stabilizing anion–π interactions within the cylindrical micropores, satisfying criterion 3.

Finally, the average slope of the insets in Figure 4d–f corresponds to a volume decrease, which is 7-fold higher than what would be expected based on the actual size of BF_4_^–^ (see details in the Supporting Information). Figure S8 shows the decrease in SSA expressed as the area occupied by the immobilization of one single molecule of **Pt**_**1**_, **Pt**_**2**_, or **Pt**_**12**_. We see that this decrease is correlated to the number of counterions per complex. For all three cases, the average micropore diameter increases from ∼0.9 to ∼1.1 nm with increasing loading of the complexes (Table S3). This further supports our anion confinement hypothesis: BF_4_^–^ anions likely block the micropores by remaining at the pore mouth and staying close to the cationic complex. The latter can also block the pore entrance and prevent N_2_ adsorption due to its size and charge.

### Adsorption Kinetics and Isotherms of the Complexes

**Figure 5:**
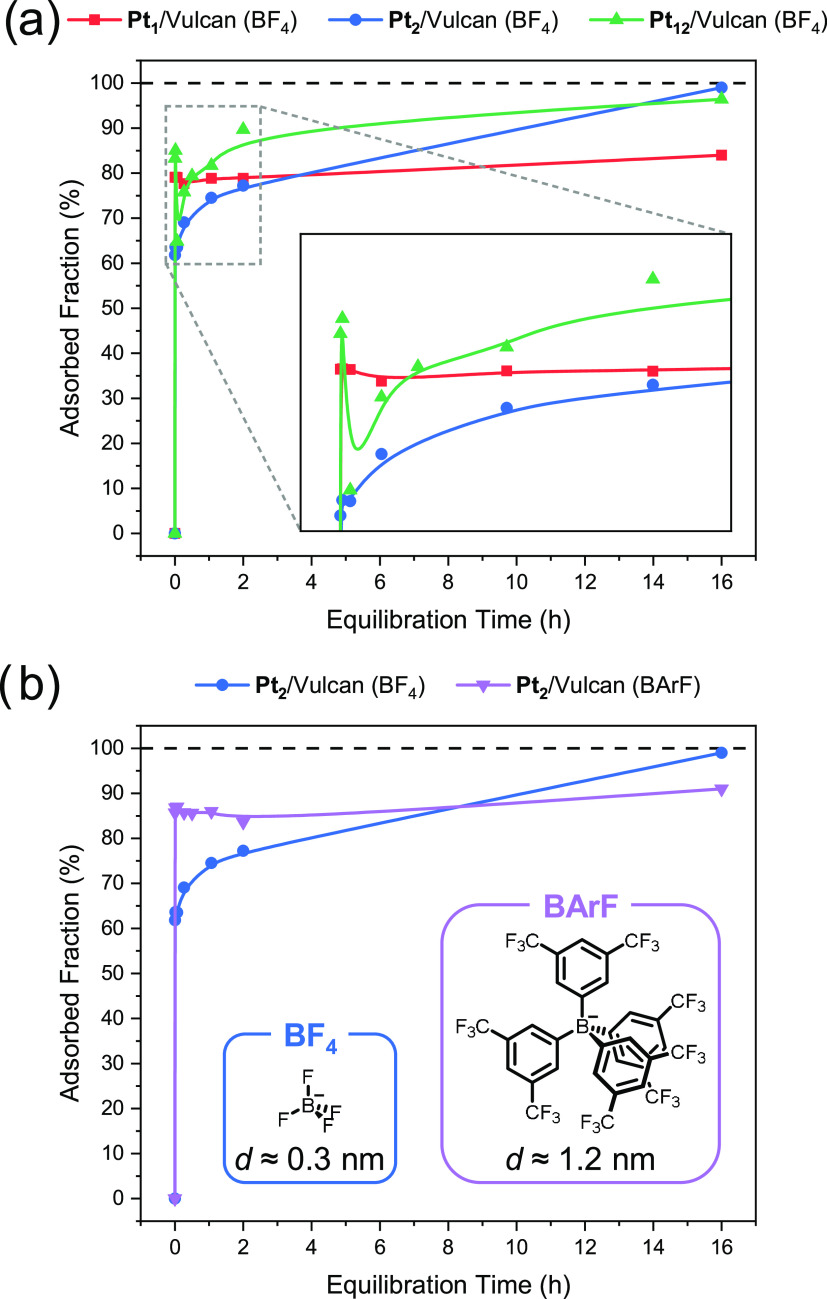
Adsorption kinetics on Vulcan of (a) **Pt**_**1**_, **Pt**_**2**_, and **Pt**_**12**_ bearing BF_4_ anions and (b) **Pt**_**2**_ bearing BF_4_ and BArF anions (5.0 μmol Pt g_Vulcan_^–1^).

Next, we monitored the immobilization process of **Pt**_**1**_, **Pt**_**2**_, and **Pt**_**12**_ on Vulcan over time by studying their binding kinetics (Figure 5a). This was done by sampling at different times after the complexes were added to a well-dispersed suspension of Vulcan. Each sample was quickly filtered, and the filtrate was analyzed to determine the adsorbed fraction (see Figures S9–S14 and further details in the Experimental Section). For **Pt**_**1**_ (red curve in Figure 5a), the surface coverage reached its final value (∼80%) within 15 s of equilibration time, indicating nonrestricted and relatively weak binding (*vide infra*). The other two complexes adsorb spontaneously on the surface in a two-step process: a fast initial adsorption of a large fraction of the dissolved complexes followed by a slow adsorption of the remaining amount. In the case of **Pt**_**2**_ (blue curve in Figure 5a), ca. 65% of the complexes adsorbed within 15 seconds, then the mixture remains stable for 4 min, and then the rest of the complexes adsorb slowly, within 16 h. The most striking adsorption behavior was observed for the largest complex, **Pt**_**12**_ (green curve in Figure 5a). Nearly 85% of it adsorbed within 15 seconds, followed by a release of 20% back into solution after 4 min and a slow readsorption thereafter to reach complete adsorption within 16 h.

To further test our hypothesis of counterion confinement in surface micropores, we synthesized a **Pt**_**2**_ complex with BArF (BArF = [{3,5-(CF_3_)_2_C_6_H_3_}_4_B]) anions. We selected BArF as a suitable counterion for this because it has a diameter of ∼1.2 nm, too large to fit in the micropores of Vulcan (*d* ≈ 0.7 nm).^30^ We studied its adsorption kinetics on Vulcan and compared it to that of its BF_4_ analogue (Figure 5b). The BArF complex shows fast initial binding after which the surface coverage (∼85%) does not change anymore. This complex has fast initial binding because surface binding can happen everywhere on the surface via π–π stacking interactions between the graphitic parts of Vulcan and the aromatic parts of BArF. The fact that full coverage is not reached within 16 h indicates weaker surface binding relative to the BF_4_ analogue, which does reach full coverage under the same conditions (Figure 5b). This implies that binding by confining BF_4_ anions in the micropores enables stronger surface binding and potentially a better dispersion of the complexes, as this binding is limited to specific sites.

Based on these results, we suggest that there are two processes here, namely, surface adsorption and surface binding. The former is fast, as it involves only van de Waals forces and can occur on any site on the surface. Nor does it distinguish much between types and sizes of complexes. The latter process, however, is slower and more intricate. It forms interactions that require suitable binding spots. Such a two-step mechanism, generally referred to as indirect adsorption, is much more common than the direct option, where the complex stays put at its initial point of adsorption.^31^ The second step involves, per definition, a detachment of the complex from its initial binding site. In the case of **Pt**_**12**_, that bears no less than 24 counterions, dissolution is more favored, and therefore the lack of suitable sites leads to an intermediate net surface desorption, until each complex finds its place.

To gain further insight into the binding strength of **Pt**_**1**_, **Pt**_**2**_, and **Pt**_**12**_ on Vulcan, we measured their adsorption isotherms (Figure S15a–c). This was done by preparing sets of samples with an equal amount of support, yet using different concentrations of the complexes, starting from 7.15 μM Pt and going up to 70.71 μM (the concentration used in the adsorption kinetics experiments). After 16 h of equilibration, the samples were filtered, and the concentrations of the supernatants were determined to quantify the adsorbed fraction (see Figures S9–S14 and further details in the Experimental Section). We see that all of the isotherms fit well to the solution analogue of the Brunauer–Emmett–Teller (BET) model (*R*^2^ > 0.996 for eight observations).^23^**Pt**_**1**_ shows a typical C1 curve, indicating unrestricted adsorption at equal binding sites.^32^ For **Pt**_**2**_ and **Pt**_**12**_ the curve is better described by a Langmuir-type isotherm, L3, indicating minor interactions between the adsorbed complexes. This difference is in line with the increase in the adsorbate–adsorbate binding constants for the larger complexes (Table S5).

Most importantly, the surface–adsorbate binding constants (*K*_S_), **Pt**_**1**_ (5.3 M^–1^), **Pt**_**2**_ (6.3 × 10^4^ M^–1^), and **Pt**_**12**_ (1.6 × 10^6^ M^–1^), increase quadratically with the number of anions per molecule (Figure S14d). We hypothesize that this increase is caused by cooperative binding of multiple anions to one single complex (just like with a multidentate ligand) satisfying criterion 1.^33^ The value of *K*_S_ for **Pt**_**12**_ corresponds to a free energy of adsorption of −35 kJ mol^–1^. This value is far beyond van der Waals or any π-interactions. It is more in the range of multiple, cooperative ionic interactions.^34^

To learn more about the adsorption of **Pt**_**1**_ and **Pt**_**2**_ on Vulcan, we also determined the values of *K*_S_ during the impregnation at *t* = 10 min and *t* = 2 h (Figures S16 and S17). For **Pt**_**1**_, we know that all adsorption happens within 15 s (red curve in Figure 5a) and we found that *K*_S_ remained constant throughout the adsorption process (*t* = 10 min, 5.2 M^–1^; *t* = 2 h, 5.4 M^–1^; *t* = 16 h, 5.3 M^–1^). This means that either the second step of travel and binding at a vacant site already occurred for all complexes within 15 s or this step does not take place at all. Conversely, **Pt**_**2**_ shows a second binding process going from *t* = 4 min to 16 h (blue curve in Figure 5a), and *K*_S_ was found to increase over time (*t* = 10 min, 5.6 × 10^4^ M^–1^; *t* = 2 h, 6.0 × 10^4^ M^–1^; *t* = 16 h, 6.3 × 10^4^ M^–1^). Thus, the average binding strength of adsorbed **Pt**_**2**_ increases during impregnation. This supports our hypothesis that the first step is a weak surface adsorption, and the second step is a strong surface binding, which is induced by anion confinement in the micropores.

### Proposed Immobilization Mechanism

**Figure 6:**
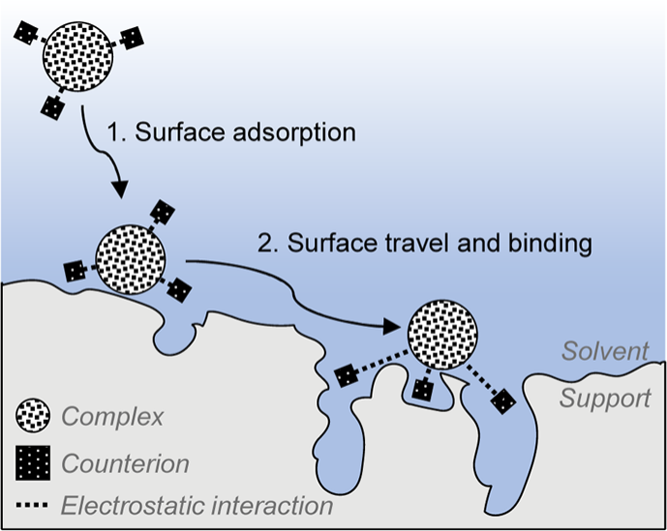
Proposed immobilization mechanism of molecular complexes on charge-neutral supports via adsorption and surface travel to a suitable binding site.

Based on the above results, we propose a two-step immobilization mechanism (Figure 6): In the first surface adsorption step, the complex adsorbs at a random site in a process driven by van der Waals interactions and the free energy gain of physisorption. Subsequently, it travels over the surface until it finds a suitable binding spot: a site where sufficient anions can confine in micropores close to the complex, resulting in surface binding.

## Conclusions

Counterion confinement in surface pores is a simple and efficient method for noncovalent grafting of complexes to charge-neutral supports. Moreover, the method is generic, as the same counterion can be used for binding different complexes of varying charges and sizes. The process comprises two steps: adsorption at a random site, followed by surface travel, and binding through counterion confinement in the micropores. Importantly, the travel across the surface promotes a homogeneous distribution of the complexes. Such a distribution is an advantage in many applications including catalysis and molecular sensing. The final binding strength depends on the number of counterions per complex: more counterions lead to stronger binding. Overall, this generic and simple method for binding complexes to neutral surfaces opens opportunities for the creation of new materials that can help address today’s environmental problems.

## Supporting Information Available

ICP-OES data, desorption studies, nitrogen physisorption studies, UV–vis calibration data, additional calculations, and full data of the adsorption isotherms (PDF)
