# Supplementary material for: Noncovalent Grafting of Molecular Complexes to Solid Supports by Counterion Confinement
Source: J Phys Chem C Nanomater Interfaces. 2023 Dec 12;127(50):24129–36. doi: 10.1021/acs.jpcc.3c05691 (PMC10749480; doi:10.1021/acs.jpcc.3c05691)
Supplement: Supplementary file 1 — jp3c05691_si_001.pdf [file jp3c05691_si_001.pdf]

Supporting Information

Non-covalent Grafting of Molecular Complexes to Solid Supports by Counterion Confinement

Petrus C. M. Laan [a], Eduard O. Bobylev [a], Norbert J. Geels [a], Gadi Rothenberg [a,*], Joost N. H. Reek [a,*] and Ning Yan [a,b,*]

[a] Van 't Hoff Institute for Molecular Sciences, University of Amsterdam, Science Park 904, 1098 XH Amsterdam, The Netherlands.

[b] Key Laboratory of Artificial Micro- and Nano-Structures of Ministry of Education, School of Physics and Technology, Wuhan University, Wuhan, 430072, China.

Table of contents

1. Inductively coupled plasma optical emission spectrometry (ICP-OES) 2
2. Desorption studies 3
3. Nitrogen adsorption – desorption studies: Rouquerol analyses 4
   Vulcan 4
   Pt_1/Vulcan 5
   Pt_2/Vulcan 6
   Pt_{12}/Vulcan 7
4. UV-Vis calibration curves 9
   Pt_1 9
   Pt_2 10
   Pt_{12} 11
5. Additional calculations 12
   Decrease in specific surface area (SSA) 12
   Decrease in micropore volume (V_{micro}) 13
6. Adsorption isotherms 14
7. References 16

1. Inductively coupled plasma optical emission spectrometry (ICP-OES)

Table S1. Pt loadings of **Pt_1**/Vulcan and **Pt_2**/Vulcan.

**Catalyst**	**Catalyst loading (μmol Pt g_{Vulcan}^{-1})**	
	**Expected**	**Observed**
**Pt_1**/Vulcan	5.0	5.5±0.5
**Pt_2**/Vulcan	5.0	5.0±0.5

2. Desorption studies

Table S2. Desorption percentages of **Pt_2**/Vulcan based on UV-Vis adsorption studies using the calibration curve in **Figure S10** and **S11** (MeCN) and previous work (DMSO).[1]

**Conditions**	**Desorption (%)**
MeCN, room temperature, 16h	0
MeCN, Soxhlet, 16h	0
DMSO, room temperature, 16h	>95

3. Nitrogen adsorption – desorption studies: Rouquerol analyses

Vulcan

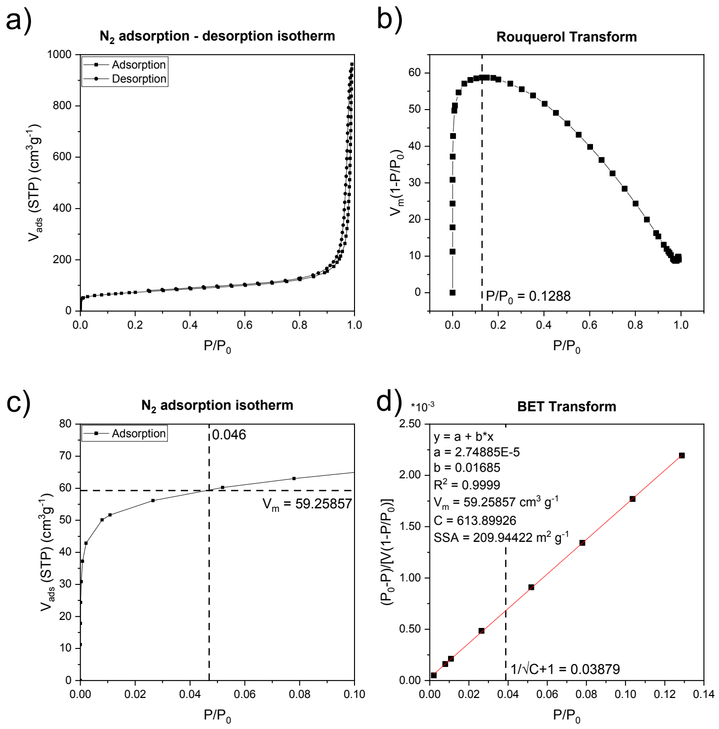

Figure S1. Porosity analysis of Vulcan. a) N_2 adsorption and desorption isotherms at 77 K; b) Rouquerol transform plot; c) zoom-in of the N_2 adsorption isotherm at 77 K and d) BET transform plot.

Pt₁/Vulcan

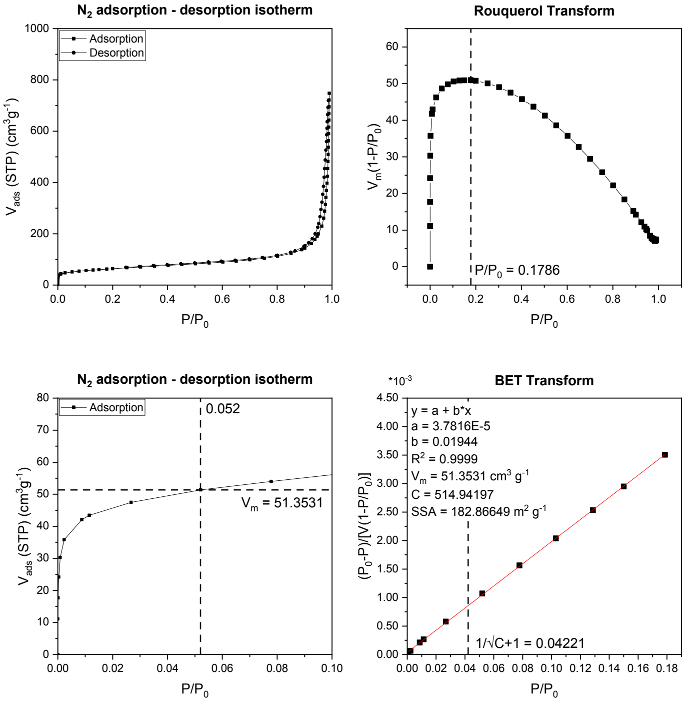

Figure S2. Porosity analysis of **Pt₁**/Vulcan (5.0 μmol Pt g_{vulcan}^{-1}). a) N_2 adsorption and desorption isotherms at 77 K; b) Rouquerol transform plot; c) zoom-in of the N_2 adsorption isotherm at 77 K and d) BET transform plot.

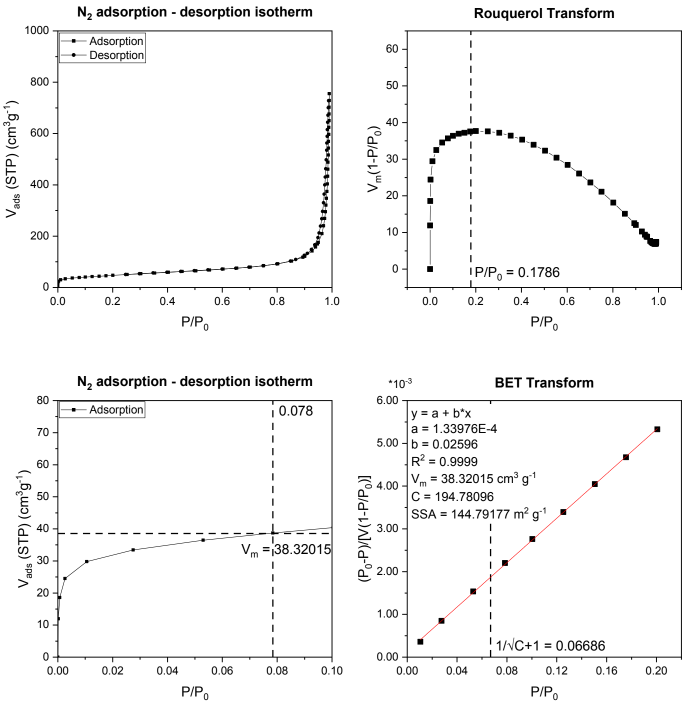

Figure S3. Porosity analysis of **Pt₁**/Vulcan (12.5 μmol Pt g_{vulcan}^{-1}). a) N_2 adsorption and desorption isotherms at 77 K; b) Rouquerol transform plot; c) zoom-in of the N_2 adsorption isotherm at 77 K and d) BET transform plot.

Pt_2/Vulcan

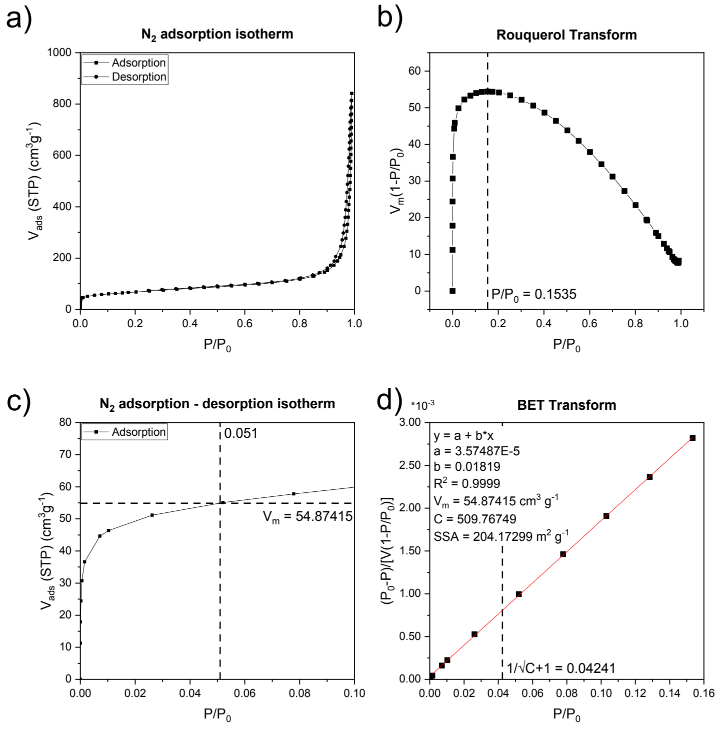

Figure S4. Porosity analysis of **Pt_2**/Vulcan (5.0 µmol Pt g_{vulcan}^{-1}). a) N_2 adsorption and desorption isotherms at 77 K; b) Rouquerol transform plot; c) zoom-in of the N_2 adsorption isotherm at 77 K and d) BET transform plot.

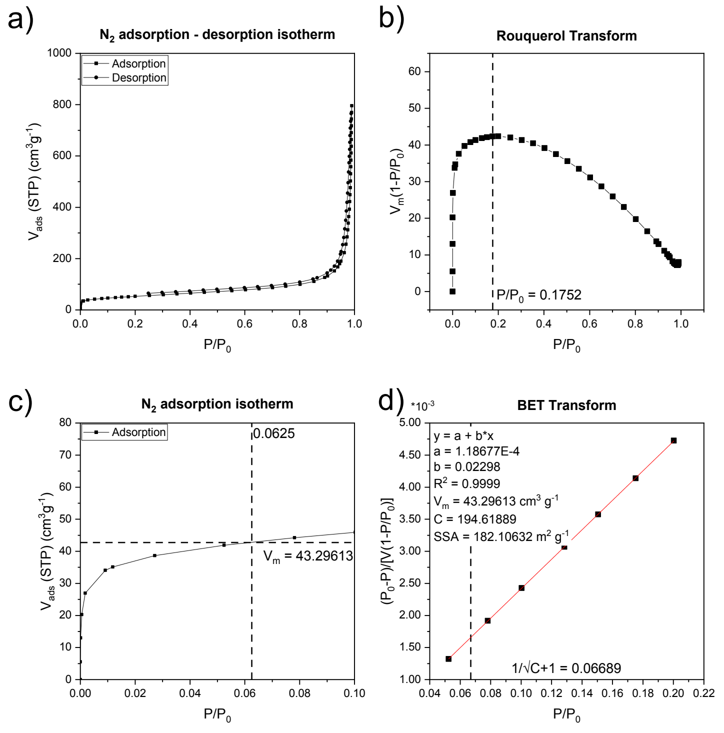

Figure S5. Porosity analysis of **Pt_2**/Vulcan (12.5 µmol Pt g_{vulcan}^{-1}). a) N_2 adsorption and desorption isotherms at 77 K; b) Rouquerol transform plot; c) zoom-in of the N_2 adsorption isotherm at 77 K and d) BET transform plot.

Pt_{12}/Vulcan

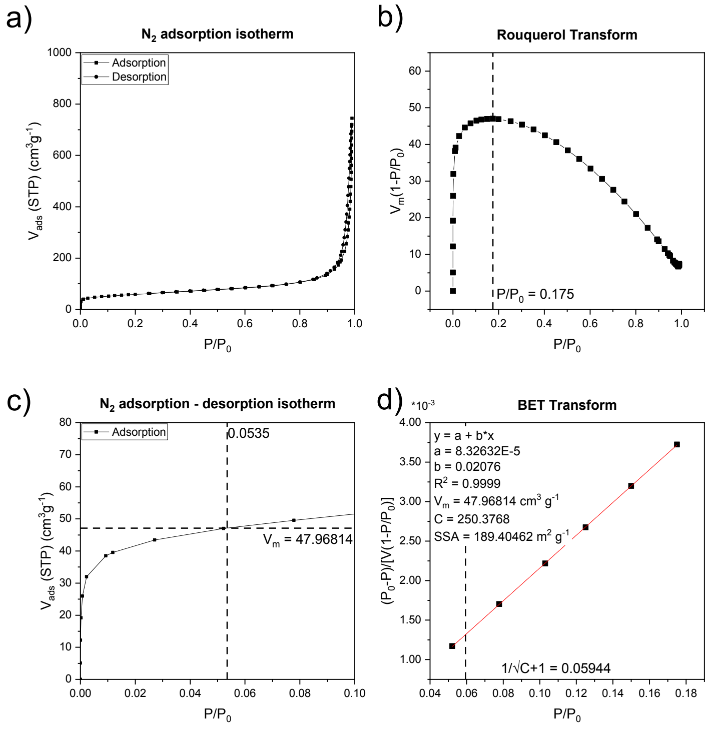

Figure S6. Porosity analysis of **Pt_{12}**/Vulcan (5.0 µmol Pt g_{vulcan}^{-1}). a) N_2 adsorption and desorption isotherms at 77 K; b) Rouquerol transform plot; c) zoom-in of the N_2 adsorption isotherm at 77 K and d) BET transform plot.

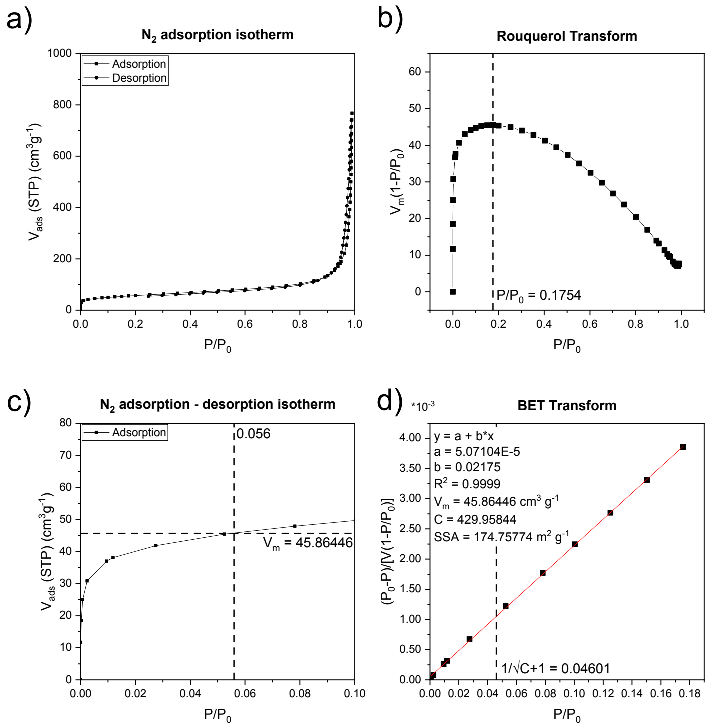

Figure S7. Porosity analysis of **Pt_{12}**/Vulcan (12.5 µmol Pt g_{vulcan}^{-1}). a) N_2 adsorption and desorption isotherms at 77 K; b) Rouquerol transform plot; c) zoom-in of the N_2 adsorption isotherm at 77 K and d) BET transform plot.

Table S3. Average micropore withs of **Pt$_1$**/Vulcan, **Pt$_2$**/Vulcan and **Pt$_{12}$**/Vulcan at different platinum loadings.

**Complex**	**Average pore with (nm)**		
	**0 µmol Pt g_{Vulcan}^{-1}**	**5.0 µmol Pt g_{Vulcan}^{-1}**	**12.5 µmol Pt g_{Vulcan}^{-1}**
**Pt$_1$**	0.95	1.06	1.19
**Pt$_2$**	0.95	0.97	1.00
**Pt$_{12}$**	0.95	0.95	1.05

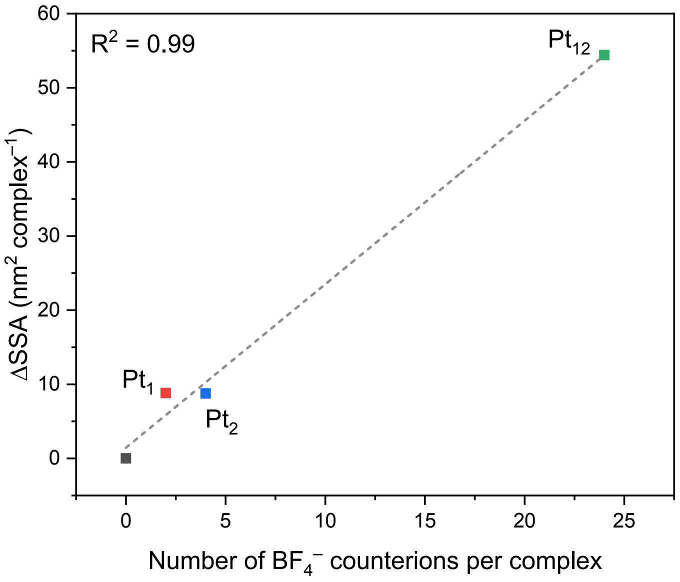

Figure S8. Decrease in specific surface area (SSA) upon the immobilization of one molecule of **Pt$_1$**, **Pt$_2$** or **Pt$_{12}$** (based on nitrogen sorption data), expressed as a function of BF_4^- ions per complex (see further details on the calculations in section 5 below).

4. **UV-Vis calibration curves**

Pt_1

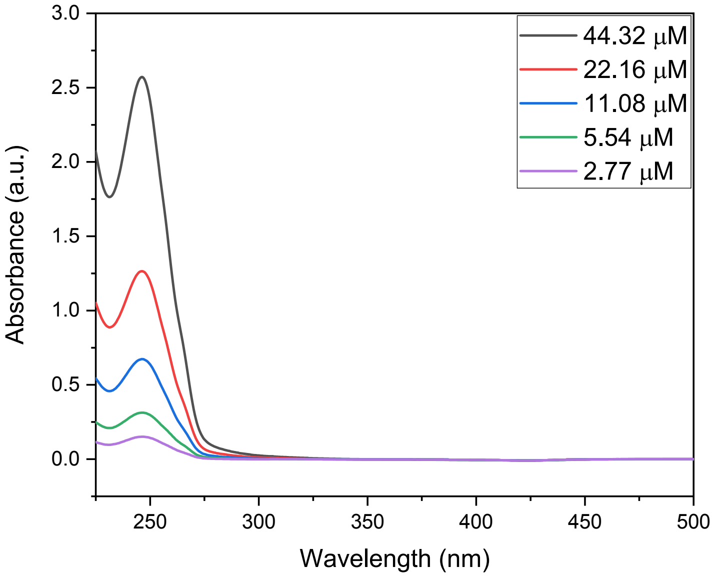

Figure S9. UV-Vis spectra of **Pt_1** in MeCN in the concentration range 2.77 – 44.32 µM measured in a cuvette with a path length of 10 mm.

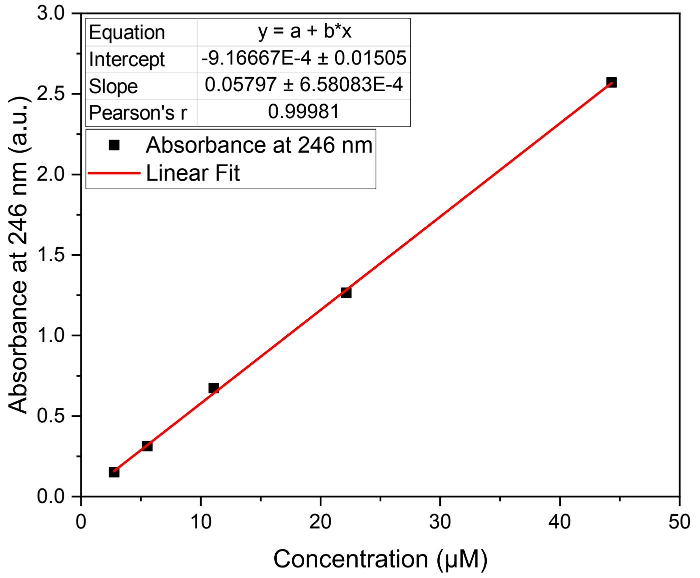

Figure S10. Lambert-Beer plot of **Pt_1** in MeCN in the concentration range 2.77 – 44.32 µM measured in a cuvette with a path length of 10 mm.

Pt_2

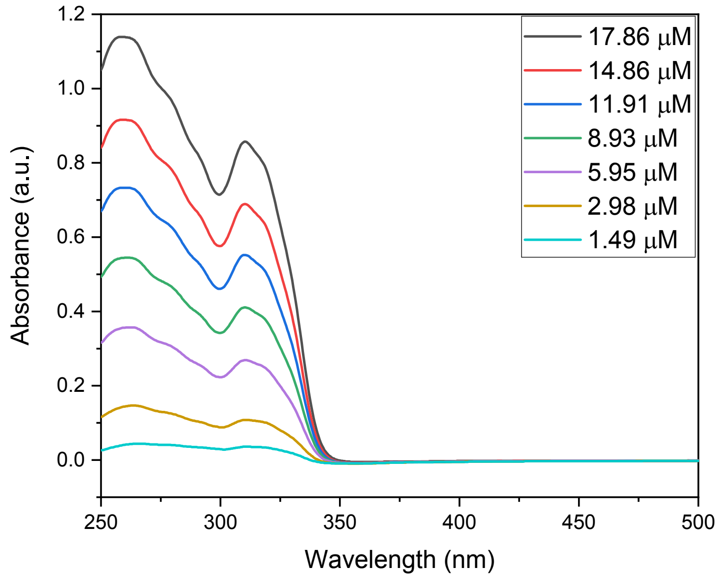

Figure S11. UV-Vis spectra of **Pt_2** in MeCN in the concentration range 1.49 – 17.86 μM measured in a cuvette with a path length of 2 mm.

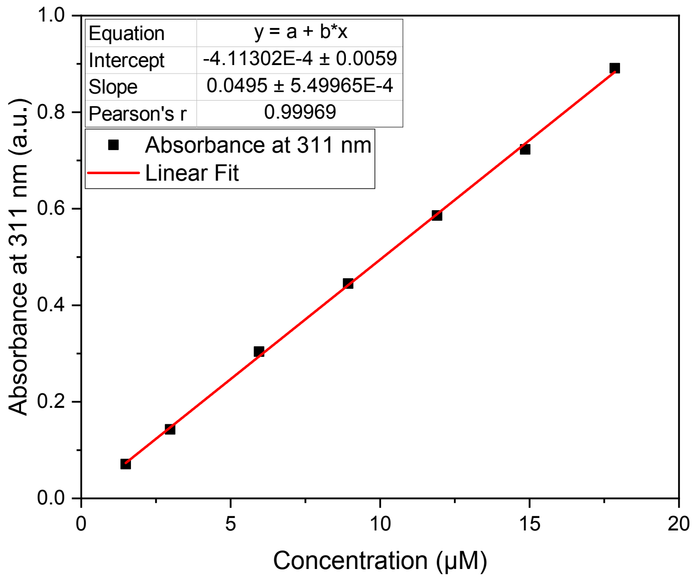

Figure S12. Lambert-Beer plot of **Pt_2** in MeCN in the concentration range 1.49 – 17.86 μM measured in a cuvette with a path length of 2 mm.

Pt_{12}

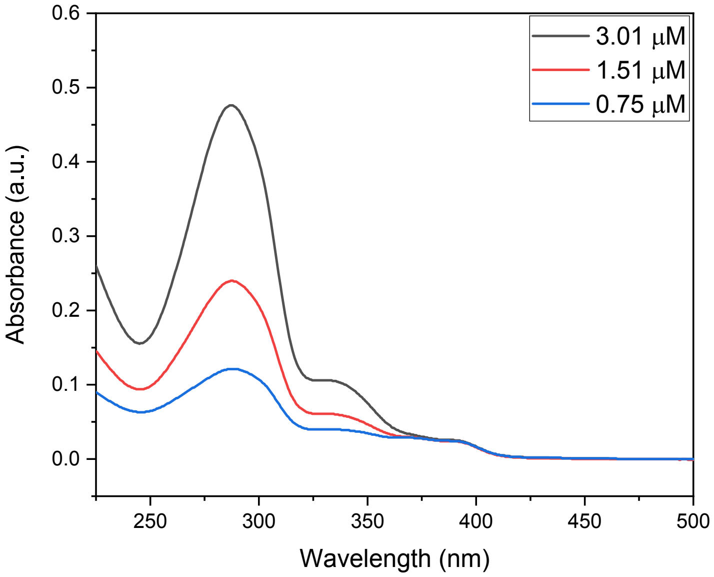

Figure S13. UV-Vis spectra of **Pt_{12}** in MeCN in the concentration range 0.75 – 3.01 µM measured in a cuvette with a path length of 2 mm.

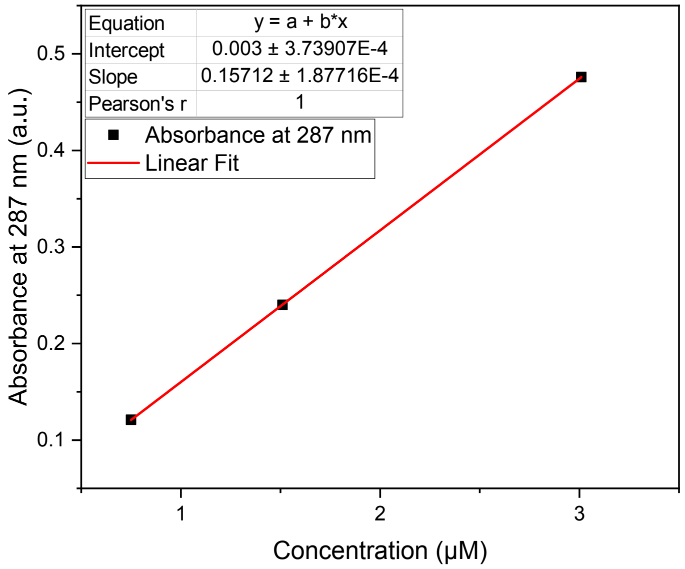

Figure S14. Lambert-Beer plot of **Pt_{12}** in MeCN in the concentration range 0.75 – 3.01 µM measured in a cuvette with a path length of 2 mm.

5. Additional calculations

Decrease in specific surface area (SSA)

The slope, ΔSSA, of the insets in **Figure 4a-c** have a unit that is described in Eq. S1.

$$\frac{\Delta y}{\Delta x} = \frac{[m^2 g^{-1}]}{[\mu mol_{Pt}\, g^{-1}]} = \frac{[m^2]}{[\mu mol_{Pt}]} \#(S1)$$

This unit can be rewritten to (nm^2 $complex^{-1}$) as described in Eq. S2 because 1 m^2 = 1*10^{18} nm^2, x number of Pt atoms = 1 complex (x = 1, 2 or 12 for **Pt_1**, **Pt_2** and **Pt_{12}**), 1 μmol = 1*10^{-6} mol and 1 mol Pt = N_A Pt atoms (N_A = 6.0221408*10^{23}).

$$Decreasing\ Area\ (nm^2 complex^{-1}) = |\Delta SSA| * \frac{10^{18} * number\ of\ Pt\ atoms\ in\ complex}{10^{-6} * N_A} \#(S2)$$

The surface area that one molecule could cover was estimated based on X-ray structures of **Pt_1**[1], **Pt_2**[1] and **Pt_{12}**[2]. The measured diameters are listed in **Table S2**. In the case of **Pt_{12}**, an X-ray structure of an isostructural cage was used because an X-ray structure of this cage is not reported to date. The diameter of the complexes was used to calculate the area that it could cover as described in Eq. S3.

$$Covering\ Area\ (nm^2 complex^{-1}) = \pi * \left(\frac{d_{complex}\,[nm]}{2}\right)^2 \#(S3)$$

This gives covering area's that are ~5 times lower than the observed decrease in surface area (**Table S4**). Note: N_2 adsorption of the complexes itself is not considered here as M_2L_4 cage structures like **Pt_2** are known to have negligible affinity for N_2 adsorption themselves.[3]

Table S4. Structural parameters of the complexes.

Complex	ΔSSA (m^2 μmol_{Pt}^{-1})	ΔSSA (nm^2 $complex^{-1}$)	$d_{complex}$ (nm)[1,2]	Covering Area (nm^2 $complex^{-1}$)	ΔSSA / Covering Area
**Pt_1**	-5.31	8.7	1.34	1.41	6.2
**Pt_2**	-2.45	8.9	1.16	1.06	8.3
**Pt_{12}**	-2.91	54.4	3.80	11.34	4.8

Decrease in micropore volume (V_{micro})

The slope, ΔV_{micro}, of the insets in **Figure 4d-f** have a unit that is described in Eq. S4.

$$\frac{\Delta y}{\Delta x} = \frac{[cm^3 g^{-1}]}{[\mu mol_{Pt}\, g^{-1}]} = \frac{[cm^3]}{[\mu mol_{Pt}]} \#(S4)$$

This unit can be rewritten to (nm^3 BF_4 $anion^{-1}$) as described in Eq. S5 because 1 cm^3 = 1*10^{21} nm^3, 1 μmol = 1*10^{-6} mol, 1 mol Pt = N_A Pt atoms (N_A = 6.0221408*10^{23}) and 1 Pt atom = 2 BF_4 anions.

$$Decreasing\ Volume\ (nm^3\ BF_4\ anioin^{-1}) = |\Delta V_{micro}| * \frac{10^{21}}{2 * 10^{-6} * N_A} \#(S5)$$

Taking the average slope, 0.00156 cm_3 mol_{Pt}^{-1}, results in an average decreased volume of 1.30 nm^3 BF_4 per anion.

The volume that one BF_4 anion would occupy can be estimated best based on its average diameter while having anion-π interactions, that is ~0.35 nm,[4] using the formula described in Eq. S6.

$$Occupying\ Area\ (nm^3\ BF_4\ anioin^{-1}) = \frac{4}{3} * \pi * \left(\frac{d_{complex}\ [nm]}{2}\right)^3 \#(S6)$$

This gives an estimated occupied area by one BF_4 anion equal to 0.18 nm^3, a value that is ~7 times lower than the observed volume decrease.

6. Adsorption isotherms

Table S5. Adsorbate–adsorbate binding constants of the complexes on Vulcan, t = 16 h.

Complex	Adsorbate–adsorbate binding constants (M^{-1})
$\mathbf{Pt_1}$	$6.4x10^2$
$\mathbf{Pt_2}$	$1.1x10^4$
$\mathbf{Pt_{12}}$	$1.1x10^5$

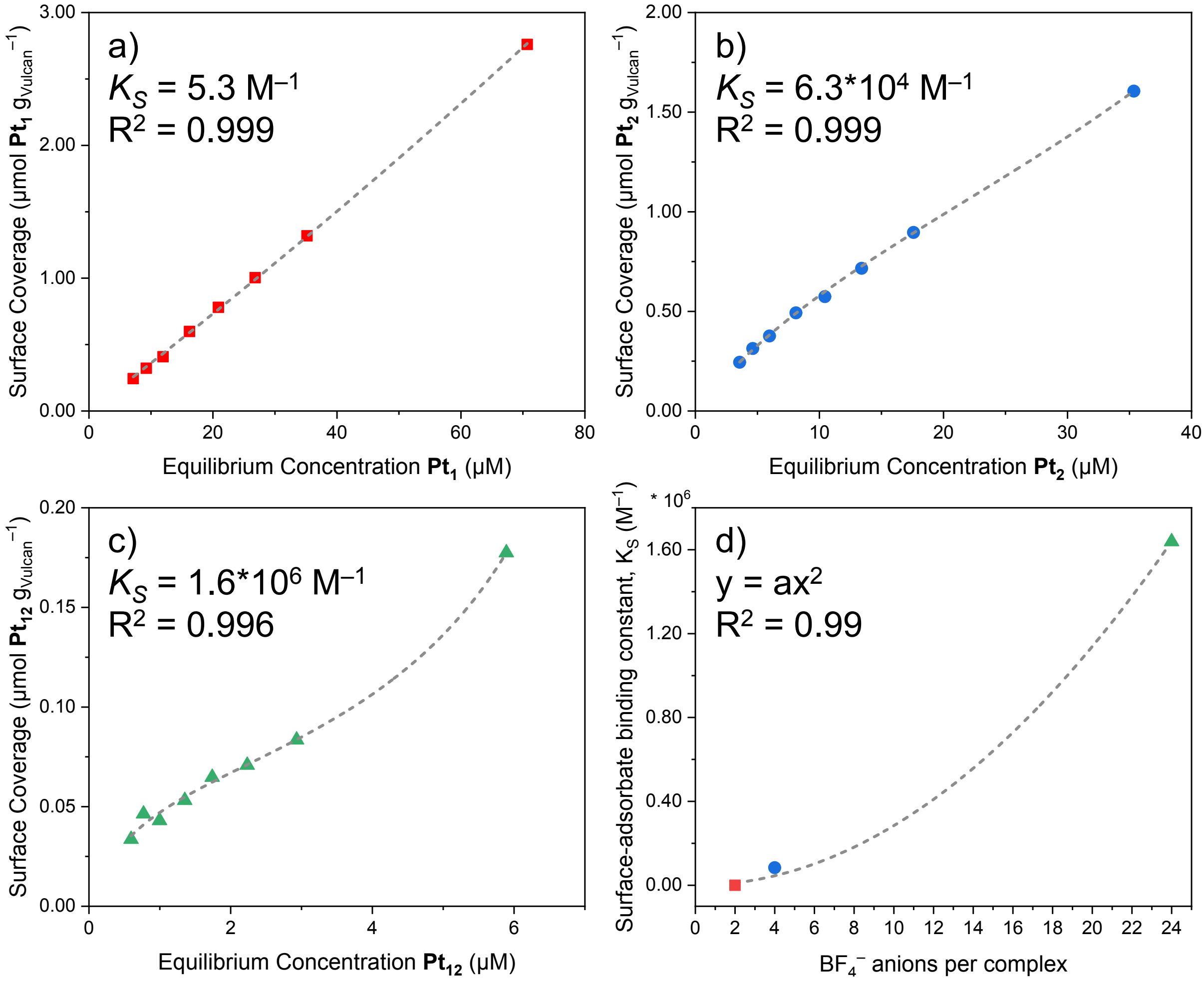

Figure S15. Adsorption isotherms of $\mathbf{Pt_1}$, $\mathbf{Pt_2}$ and $\mathbf{Pt_{12}}$ on Vulcan. a-c) Adsorption isotherms and the corresponding fitting of the solution analogue of the Brunauer–Emmett–Teller model and d) determined surface-adsorbate binding constants K_S as a function of the number of BF_4^- anions per complex and the corresponding second order polynomial fit.

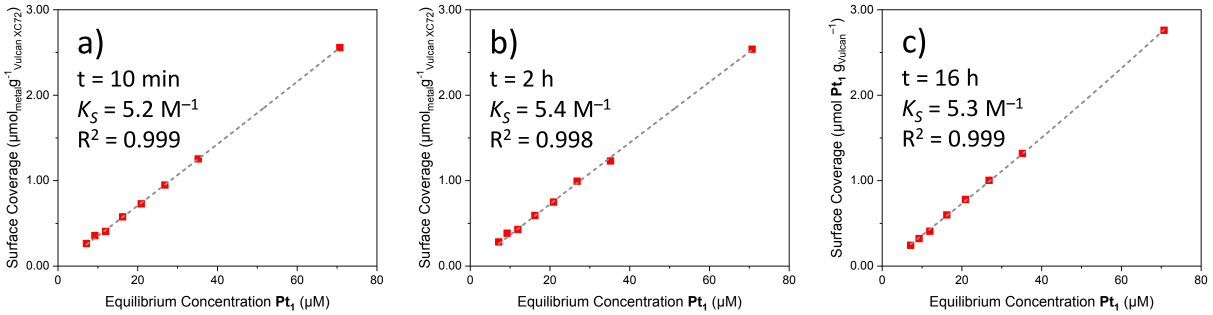

Figure S16. Adsorption isotherms of **Pt_1** on Vulcan. Adsorption isotherms and the corresponding fitting of the solution analogue of the Brunauer–Emmett–Teller model after a) 10 min, b) 2 h and c) 16h of equilibration time.

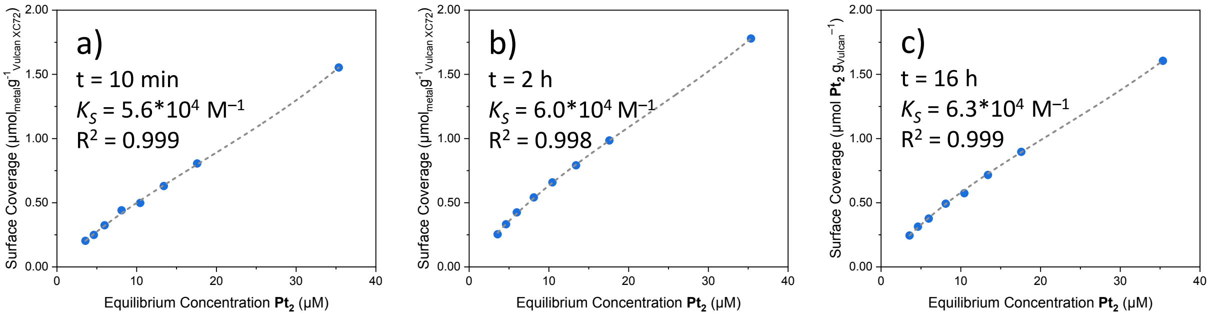

Figure S17. Adsorption isotherms of **Pt_2** on Vulcan. Adsorption isotherms and the corresponding fitting of the solution analogue of the Brunauer–Emmett–Teller model after a) 10 min, b) 2 h and c) 16h of equilibration time.

7. References

(1) Laan, P. C. M.; Bobylev, E. O.; de Zwart, F. J.; Vleer, J. A.; Troglia, A.; Bliem, R.; Rothenberg, G.; Reek, J. N. H.; Yan, N. Tailoring Secondary Coordination Sphere Effects in Single-Metal-Site Catalysts by Surface Immobilization of Supramolecular Cages. *Chem. Eur. J.* **2023**, e202301901. https://doi.org/10.1002/chem.202301901.

(2) Yokoyama, H.; Ueda, Y.; Fujita, D.; Sato, S.; Fujita, M. Finely Resolved Threshold for the Sharp M12L24/M24L48 Structural Switch in Multi-Component MnL2n Polyhedral Assemblies: X-Ray, MS, NMR, and Ultracentrifugation Analyses. *Chem. Asian J.* **2015**, *10* (10), 2292–2295. https://doi.org/10.1002/asia.201500519.

(3) Chen, L.; Yang, T.; Cui, H.; Cai, T.; Zhang, L.; Su, C.-Y. A Porous Metal–Organic Cage Constructed from Dirhodium Paddle-Wheels: Synthesis, Structure and Catalysis. *J. Mater. Chem. A* **2015**, *3* (40), 20201–20209. https://doi.org/10.1039/C5TA05592J.

(4) Bauzá, A.; J. Mooibroek, T.; Frontera, A. Towards Design Strategies for Anion–π Interactions in Crystal Engineering. *CrystEngComm* **2016**, *18* (1), 10–23. https://doi.org/10.1039/C5CE01813G.
